# Cataloging of Cd Allocation in Late Rice Cultivars Grown in Polluted Gleysol: Implications for Selection of Cultivars with Minimal Risk to Human Health

**DOI:** 10.3390/ijerph17103632

**Published:** 2020-05-21

**Authors:** Qiang Lin, Wenbin Tong, Bilal Hussain, Yasir Hamid, Min Lu, Zhenli He, Xiaoe Yang

**Affiliations:** 1 Ministry of Education (MOE) Key Laboratory of Environmental Remediation and Ecosystem Health, College of Environmental and Resources Science, Zhejiang University, Hangzhou 310058, China; linqdeyx@163.com (Q.L.); bilalcheema74@gmail.com (B.H.); yasirses2007@gmail.com (Y.H.); lumin_1994@163.com (M.L.); 2 Technical Extension Station of Soil Fertilizer and Rural Energy, Qujiang, Quzhou 324022, China; 3 Indian River Research and Education Center, Institute of Food and Agricultural Sciences, University of Florida, Fort Pierce, FL 34945, USA; zhe@ufl.edu

**Keywords:** cadmium, accumulation, genotypic, translocation, contaminated soil

## Abstract

Cadmium (Cd) is a toxic trace metal that has polluted 20% of agricultural land in China where its concentration exceeds the standards for Chinese farmland. Plants are capable of accumulating Cd and other trace metals, but this capacity varies with species and cultivars within a species. Rice is a staple food consumed by half of the global population. In order to select safe late rice cultivars that are suitable late rice cultivars that can be cultivated in for growing in slightly contaminated soil, a two-year field experiment was conducted with 27 in the first year and 9 late rice cultivars in the second year. The results showed that plant Cd concentrations varied among the cultivars, with high magnitudes of variation occurred in straw and grains. Five genotypes including LR-12, LR-17, LR-24, LR-25 and LR-26 were identified as low accumulators for the first year while LR-15 and LR-17 were identified as promising cultivars based on Cd concentration in the polished rice grains (<0.02 mg kg^−1^ DW). In addition, these cultivars had favorable traits, including mineral nutrition and grain yield. Therefore, these genotypes should be considered for cultivation in slightly or moderately Cd contaminated soils.

## 1. Introduction

Rapid industrialization and hazard dumping of disposal wastes have enhanced the contamination of potential toxic metals (PTMs) to soils, causing public concerns about crop production, food safety and human health [1,2,3]. Among the PTMs, cadmium (Cd) is highly toxic and results in widespread pollution due to its extensive distribution and mobility in the environment [4,5]. As it is non-biodegradable, Cd can persist in soil for a long time [6]. According to the latest survey, Cd ranks first with 7% contamination nationwide in China and is the most commonly detected trace metal in soils [3] of the investigated sites. Both natural processes [7,8] and anthropogenic inputs contribute to PTM contamination to agricultural lands of China [3,9]. Meanwhile, uptake and accumulation of PTMs in crop plants and agricultural products affect directly the quality and productivity of crops and indirectly the human health via food chains [10].

Among all the PTMs, Cd is associated with severe health risks to organisms [11] as its intake can cause various disorders in human body (i.e., hypertension, cardiac failure, cancer, serious damage in lungs, in eyes, renal dysfunction and osteoporosis) [12]. In Asian countries, rice is considered as the major source for Cd toxicity in humans [3]. High Cd concentrations in rice grain (>0.2 mg kg^−1^) in China are mainly attributed to the local anthropogenic activities [13]. Metal intake and exposure at toxic levels occur in humans through food consumption of crops (wheat, rice, maize or derived products) grown on contaminated soils [7] and long-term consumption of contaminated food and water even at relatively low concentrations could lead to chronic diseases, including cancer [7,8].

An elevated world population has led to increased food requirement, which is not possible to achieve without using all the available sources. Several remedies are reported in the literature to cope with metal contamination, including soil washing, phytoremediation, electro-kinetics, soil flushing and vitrification [14]. However, these practices are deployed with some pros and cons, especially time and costs, which limit their application at a large scale. Moreover, the soils with slight or moderate contamination have too large an area to be interrupted for remediation without agricultural activities [6]. To meet the basic food requirements it is imperative to find alternative ways to reduce metal transfer from soil to crops grown on slightly and moderately contaminated soils. It is necessary to identify crop cultivars with low accumulative capacity for PTMs and adequate yield. This approach may provide a long term solution to the management of contaminated soil.

Genetic variability includes the difference in uptake, translocation and accumulation of PTMs among the cultivars within the same species [15]. The variation among different crops and species for PTMs uptake and accumulation have been documented in maize [16,17], wheat [18], vegetables [19], Chinese cabbage (*Brassica pekinensis* L.) [20], water spinach (*Ipomoea aquatica* Forsk.) [21] and soybeans [22]. However, minimal information is available regarding the screening of late rice to achieve minimum Cd accumulation in edible parts (polished rice) and to maximize accumulation in straw, which can remove (phytoremediation).

Rice is an important staple food crop in Asian countries, especially in China. Rice plants can absorb and accumulate more PTMs, as compared to the other crops [23]. The variations in trace metal uptake, translocation and accumulation in edible parts of late rice cultivars were poorly understood. This two-year field experiment was designed to investigate metals accumulation of 27 late rice cultivars widely grown in Zhejiang, China in the 1st year and prescreened 9 cultivars in the 2nd year. The overall objective of this study was to select high yield late rice cultivars with least Cd accumulation in edible parts (polished rice) but maximum Cd accumulation in straw in order to meet the requirement of producing safe food on lightly and moderately contaminated soils.

## 2. Materials and Methods

### 2.1. Experimental Site and Characterization

**Figure 1:**
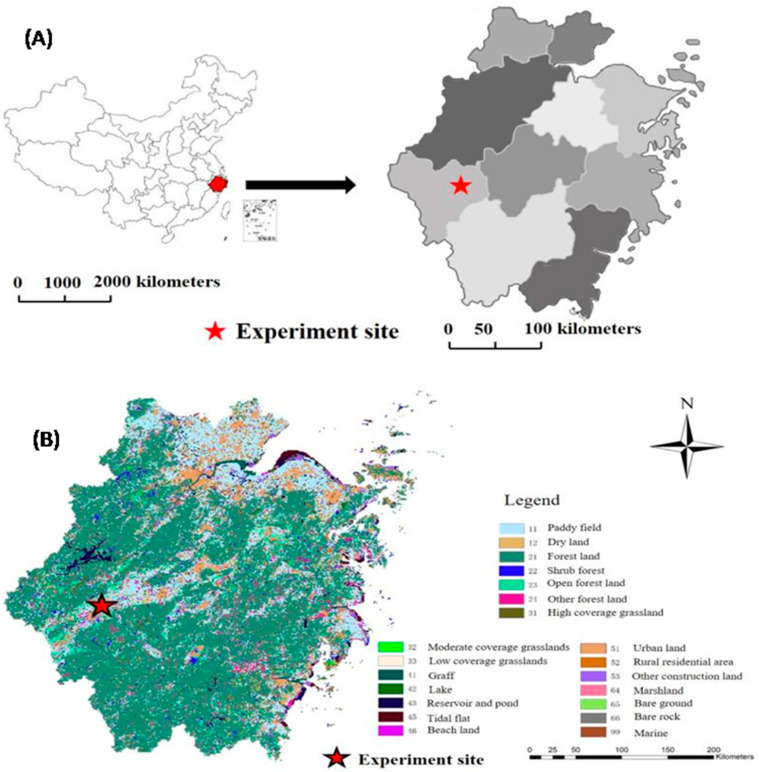
Geographical (**A**) and meteorological map (**B**) of the experimental site.

A two-year field trial was conducted in the east of Zhejiang province, China (Latitude 28°56′52″ N and longitude 118°56′30″ E) where is dominated by a subtropical monsoon climate with 18.6, 18.2 °C mean annual temperature, mean annual rainfall of 1308 and 1481 mm and sunshine of 1735 and 1748 h, respectively in the 1st and 2nd year [24]. The geographical and meteorological map of the experimental site is presented in Figure 1.

The soil was Cd contaminated mainly due to the parent materials [8], with a lower Cd concentration in the surface than that the middle and bottom layer. In addition, non-point source pollution might also contribute to the amount of soil Cd. It was estimated that inputs of atmospheric deposition, irrigation, straw return and fertilization amounted to 3.15, 4.35, 4.5 and 0.75 g/ha per year, respectively [24]. Prior to the start of experiment, basic soil physiochemical properties were analyzed [25,26] and presented in Table 1.

### 2.2. Plant Material and Sample Collection

Seeds of 27 late rice cultivars were purchased from the Quzhou seed market (Table S1). The seeds were surface sterilized, germinated and nursery-cultured in early July. The seedlings of 28 days old from the nursery were transplanted to field. The plot area was 22 m^2^ (2 m × 11 m) and each cultivar had three random replications following the complete randomized block design (RCBD). Fertilizer application (N-P_2_O_5_-K_2_O: 145-60-165 kgha^−1^) and agronomic practices were the same for all the blocks. For the second-year experiment, 9 prescreened cultivars from the first year experiment based on metal accumulation were selected as high, medium and low accumulator to verify the selection. Six plants were harvested at the maturity stage and yield attributes were measured from 1 m^2^/plot. Plant samples were rinsed with running deionization water, while roots, above-ground tissues and grains were separated and washed carefully with deionization water. The roots were first soaked in 20 mM EDTA-2 Na for 15 min and then rinsed thoroughly with DI water. All the plant samples were oven-dried at 65 °C until constant weights were achieved, while the rice grains were air dried.

### 2.3. Soil and Plant Samples Analysis

Soil samples at the depth of 0–20 cm were collected prior to transplanting and immediately after harvesting. All the samples were air dried, ground and sieved through a 1-mm sieve for basic chemical analysis. Total Cd concentration was determined by digesting 0.2 g soil with HNO_3_: HClO_4_: HF (5:1:1 v/v/v) and the supernatant was made up to volume with DI water and subjected to Inductively Coupled Plasma Massspectrometry (ICP-MS) analysis for metals concentration [11]. The plant samples (roots, shoots and grains) were weighed (0.2 g) and digested with (HNO_3_: H_2_O_2_: 5:1 v/v), the digested solution was diluted up to mark (25 mL) upon cooling and the concentration of Cd and the other elements were determined using ICP-MS (Agilent 7500a, USA, Thermo Fisher Scientific 7400, USA). To assure the quality of trace element analysis, blank and standard sample of soil (GSS-5) and plant (GBW (E) 080684) were prepared and analyzed.

Phytic acid contents were analyzed following the protocol of Dai [27]. The detailed procedure of phytic acid and protein content can be found elsewhere [3].

### 2.4. Translocation and Bioaccumulation Factor

Translocation factor (TF) was calculated the following formula to evaluate the capability of late rice cultivars in translocating Cd from root to the shoot and from shoot to grains.

(1) $$Translocation\;factor_{R-S}\left(\mathrm{TF}\right)=\frac{Cd\;conc.in\;shoot}{\mathrm{Cd}\;\mathrm{conc}.\mathrm{in}\;\mathrm{root}}$$

(2) $$Translocation\;factor_{S-G}\left(\mathrm{TF}\right)=\frac{Cd\;conc.in\;grains}{\mathrm{Cd}\;\mathrm{conc}.\mathrm{in}\;\mathrm{shoot}}$$

where (R–S) represents root to shoot ratio of Cd concentration while (S–G) represents shoot to grains ratio of Cd concentration on the DW basis.

Cadmium bioaccumulation factor was calculated by the ratio of Cd concentration in the polished rice to that in the soil.

### 2.5. Statistical Analysis

Statistical and hierarchical cluster were performed using the SPSS 20.0 (IBM, Armonk, NY, USA) and figures were prepared using Origin pro 8.5 (OriginLab Corporation, Northampton, MA, USA). All the presented data are means of three replicates ± standard error. The difference in Cd concentration among the genotypes was analyzed using one-way ANOVA at *p* < 0.05. The classification groups of cultivars were divided by different Euclidean distances; lesser Euclidean distances mean more similar individuals.

## 3. Results and Discussion

### 3.1. Plant Biomass and Grains Yield of Rice Cultivars

**Figure 2:**
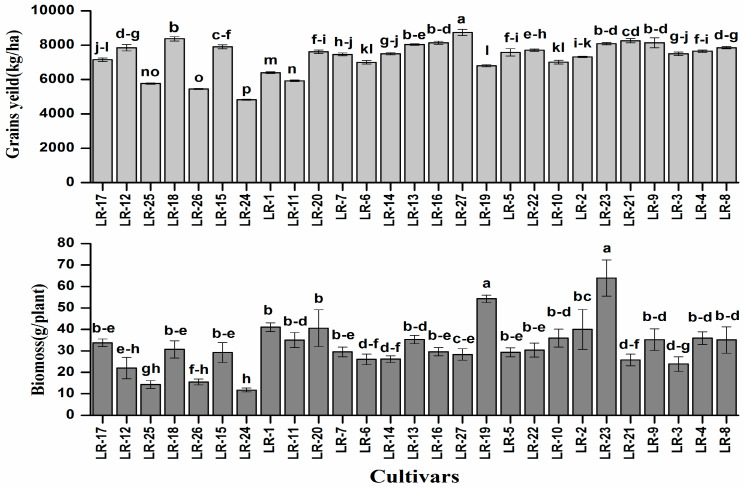
Grain yield and biomass of 27 late rice genotypes grown on Cd contaminated soil. Presented data are means of three replicates, error bars represent standard error. Different letters at the top of bars represent significant difference among the cultivars at *p* < 0.05.

Despite genetic variability, all the rice cultivars showed normal growth without any visual symptoms of Cd toxicity. However, there were considerable differences in plant biomass and grain yield (Figure 2).

Plant biomass of all the genotypes varied from 12.5 (LR-24) to 65.4 g (LR-23), with the average value of 31.3 g and a 5.23-fold difference between the highest and the lowest biomass yield. The highest grain yield was recorded at 8741 kg/ha (LR-27), which is 1.81-fold higher than the lowest yield of 4825 kg/ha (LR-24). The considerable difference among the cultivars is analogous with the results reported for maize [28,29]. The variation among the cultivars in terms of plant biomass and grain yield might be attributed to the light interception, a key factor for the production of plant biomass via photosynthesis [10,30]. It is reported that metal tolerance of the plants can be estimated by growing the varieties with high tolerance capability on the slightly to moderately contaminated soil, while tolerance is evaluated by plant biomass and grain yield [20]. The relationship between different traits or cultivars is the main feature that assists for the selection in the breeding program of rice and other crops [31,32].

### 3.2. Cadmium Concentration in Plant Tissues

**Figure 3:**
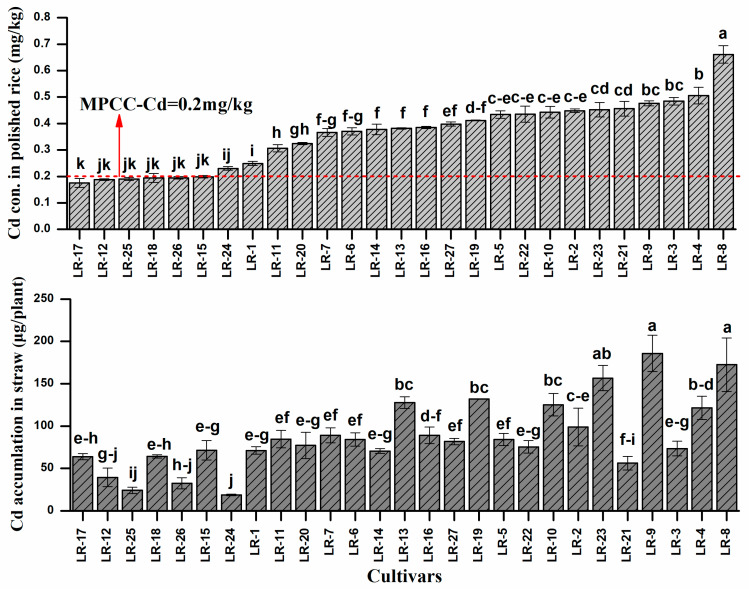
Cadmium concentration in polished rice grain and accumulation in straw of 27 late rice genotypes grown on Cd contaminated soil. Presented data are means of three replicates: error bars represent standard error. Different letters at the top of bars represent significant difference at *p* < 0.05. MPCC denotes Maximum permissible concentration in China.

Cultivars differed remarkably in Cd concentration of polished rice (Figure 3).

The distribution of Cd in different parts of plant varied among the cultivars and generally decreased in the order of root > shoot > husk > polished rice. The lowest Cd concentration in polished rice grains was 0.17 mg kg^−1^ occurred in LR-17 while the highest Cd concentration was 0.66 mg kg^−1^ measured in LR-8. The average Cd concentration in rice grain for the 27 genotypes was 0.37 mg kg^−1^, with a 3.47-fold difference between the highest and the lowest genotype. Only five cultivars, i.e., LR-17, LR-12, LR-25, LR-18 and LR-15 had a grain Cd concentration below the maximum permissible concentration in China (MPCC, 0.2 mg kg^−1^), and were considered suitable for safe production on the slightly or moderately contaminated soils. While, Cd concentration in rice straw ranged from 22.3 (LR-24) and 179.5 µg plant^−1^ (LR-9), with 8.05-fold difference between the highest and lowest accumulative cultivar. The concentration of Cd in straw is very important for the selection of cultivars not only for safe production but also for phytoremediation of contaminated soils [33]. The results from the second-year experiment revealed that the selected low accumulator (LR-12, LR-15 and LR-17) had grain Cd concentrations below the MPCC critical level for safe production (Table 2).

These results indicated the variation in plant biomass and Cd concentration among the different cultivars, with noticeable variation of Cd concentration in straw and polished rice grains of the same cultivars. The distribution ratio of Cd in the aboveground parts of the plant plays a major role in the variation of grain metal concentration. So, the difference in the grain metal concentration of the different cultivars may arise from the variation of total metal uptake. Previous studies have reported a positive correlation between grain and root concentration of metals [34,35]. Plant activities and characteristics, e.g., root surface area, mycorrhization and transpiration rate affects the metal availability in soil and subsequent accumulation in plants [36]. It was stated that, different plant species and cultivars show different trends in root activities, which ultimately affects metal availability in soil [37]. Lately, Zeng [38] also reported the consistent results that Cd concentration differs by 9.1 fold among the rice cultivars. The variance in Cd uptake and accumulation in different cultivars may be associated with root oxidation ability [39]. Previous experiments have revealed the behavioral variations of species in metal uptake and accumulation. These results reported the distribution of metals in various parts of plants, including roots and the aboveground parts [40,41].

### 3.3. Variation in Accumulation, Translocation and Bioaccumulation of Cd among the Rice Cultivars

**Figure 4:**
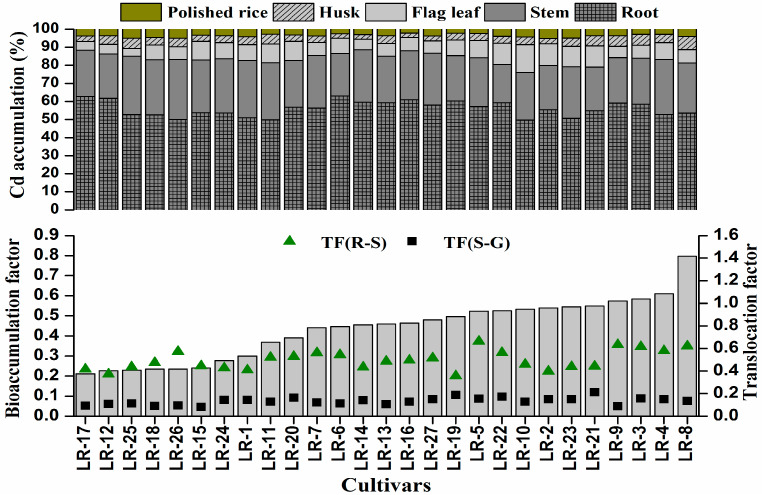
Cadmium accumulation in different parts of plant, bioaccumulation factor in polished rice grain and translocation factor of 27 late rice genotypes grown on contaminated soil. Where TF(R-S) and TF (S-G) represents root to shoot and shoot to grain translocation, respectively.

All the rice genotypes showed variation in the accumulation and translocation of metals (Figure 4). The accumulation of trace metals in roots of rice cultivars differed greatly, with smaller variations occurring in shoots and grains. Overall, Cd accumulation in different parts of rice plants followed the trend of roots > straw > polished grains. Total Cd accumulation in the whole rice plant ranged from 5.54 (LR-17) to 14.18 mg kg^−1^ (LR-9). The translocation factor (TF) was used to estimate the ability of plants to translocate metals to various parts. The TF_RS_ and TF_SG_ represents root to shoot and shoot to grains respectively and is presented in Figure 4. The maximum TF_RS_ occurred in species LR-5 (66.23%) while LR-17 had the lowest translocation (34.79%). Meanwhile, TF_SG_ was lowest in cultivar LR-16, which ranged from 9.43% (LR-16) to 22.31% (LR-21), implying a large variation among the late rice genotypes in the ability of translocating Cd from shoot to grain. Metal translocation is an important factor in controlling metals accumulation in edible parts of plants. Previous studies reported that most of the accumulated Cd in rice grains was transported via phloem [42,43]. Plant species grown on Cd contaminated soil differs in bioconcentration and translocation [44]. The differences among the rice cultivars in Cd uptake may be related to the characteristics of root absorption and exudate release. The translocation difference might also be attributed to the existing forms of Cd in soil solution. However, the mechanisms that control the translocation of Cd need further studies.

The bioaccumulation factor of Cd was lower than 1 for all the late rice cultivars with the minimum (0.2) in LR-17 and maximum bioaccumulation (0.8) in LR-8 (Figure 4). Our results showed that five cultivars had grain Cd below the MPCC (0.2 mg kg^−1^), indicating that these cultivars accumulated the least Cd in polished rice and hence were suitable for cultivation on the contaminated soil.

### 3.4. Correlation between Cd Concentration in Polished Rice and Cd Concentration in Other Parts of Plants

**Figure 5:**
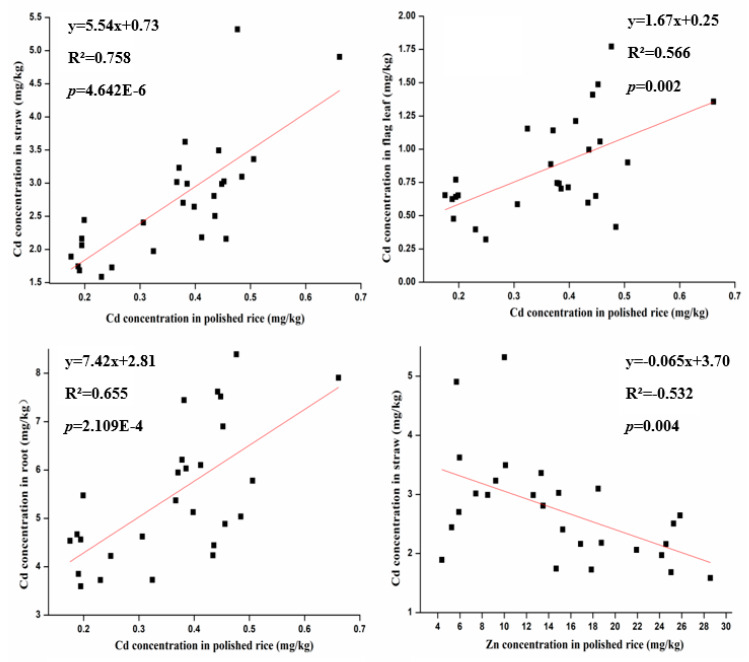
Linear correlations between Cd, Zn concentration in polished rice and Cd concentration in different parts of the plant.

There were good correlations between the Cd concentration in polished rice and that in root, straw or flag leaf (Figure 5).

The correlation coefficients (r^2^) were 0.655, 0.758 and 0.566, respectively. Whereas correlation was negative between straw Cd and Zn concentration in polished rice (R^2^ = −0.532). Previous reports showed that Zn concentration was negatively correlated with other trace metals in rice plants [3]. Our results suggested that there is an antagonism effect between Zn and Cd accumulation in polished rice, and thus application of Zn may reduce the accumulation of Cd in rice grains for production of safe food.

### 3.5. Genotypic Classification of Late Rice Cultivars Based on Cd Accumulation

**Figure 6:**
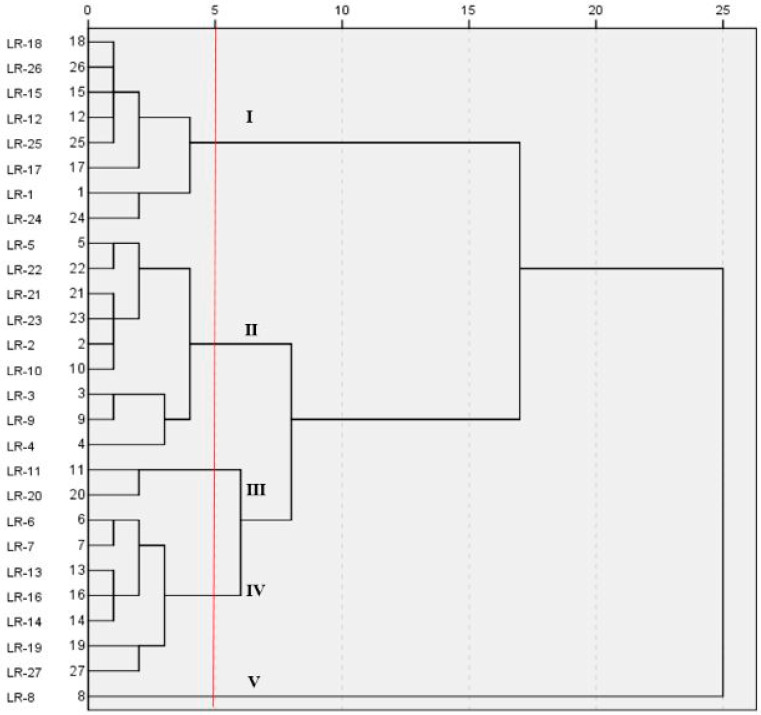
Cluster pattern of late rice cultivars on the basis of Cd accumulation in polished rice.

Genotypic classification of cultivars based on the Cd concentration was analyzed by the hierarchical cluster analysis with Euclidean distance using the Ward’s method. The hierarchical cluster analysis was used to predict the cultivars with same accumulation characteristics on the basis of Cd accumulation in the polished rice (Figure 6).

The concentration of Cd in polished rice was classified into five groups at five Euclidean distances. The cultivars LR-18, LR-26, LR-15, LR-12, LR-25, LR-17, LR-24 and LR-1 fell in the same group and were considered as low accumulators since their grain Cd concentration was below the permissible limit (0.2 mg kg^−1^).

The variation in the metal uptake in different cultivars maybe due to different acquisition capabilities of root and varies in ability to uptake and transfer [45], translocation and plant metabolic characteristics [46]. Nan [47] reported that various environmental factors could affect the bioavailability and metal accumulation in plants. Some studies reported that soil pH, cation exchange capacity (CEC), organic matter (OM) and soil texture could be important for the metal bioavailability and uptake by the crop plants [48,49]. Thus, further studies are needed to investigate the effects of soil physic-chemical properties on metal accumulation in plants, especially in the edible parts.

### 3.6. Selection Criteria for the Safe Cultivar

**Figure 7:**
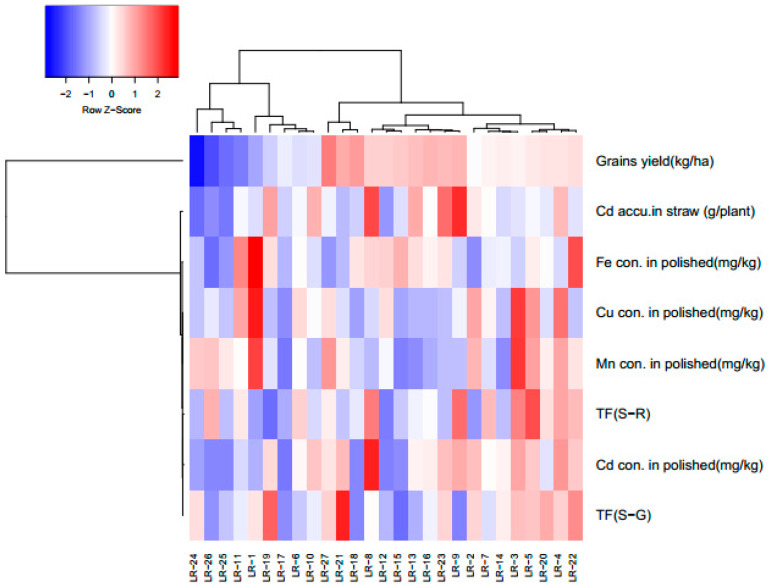
Criteria for the selection of safe production of late rice cultivars.

Genotypic difference in grain Cd accumulation provides the possibility to screen safe cultivars for production on the contaminated soils. Several factors need to be considered in screening safe or low accumulator (grains) cultivars [20], including low Cd accumulation in edible parts, TF lower than 1 and no Cd toxicity. For the present research we used the following standards for screening of low accumulator or safe cultivars: (i) polished rice Cd concentration <0.2 mg kg^−1^, (ii) high Cd accumulation in straw, (iii) TF < 1.0 and (iv) high grain yield and essential nutrients. After the 1st screening of cultivars, we divided the cultivars into three groups as high, medium and low Cd accumulator on the basis of the above standards as described in the heat map (Figure 7).

In the second year, the screening results showed that the cultivars LR-15 and LR-17 fulfilled the selection criteria with higher grain yield and mineral nutrient concentration and low Cd accumulation in polished rice (Table 2).

### 3.7. Health Risk Assessment Associated with Cd Ingestion

**Figure 8:**
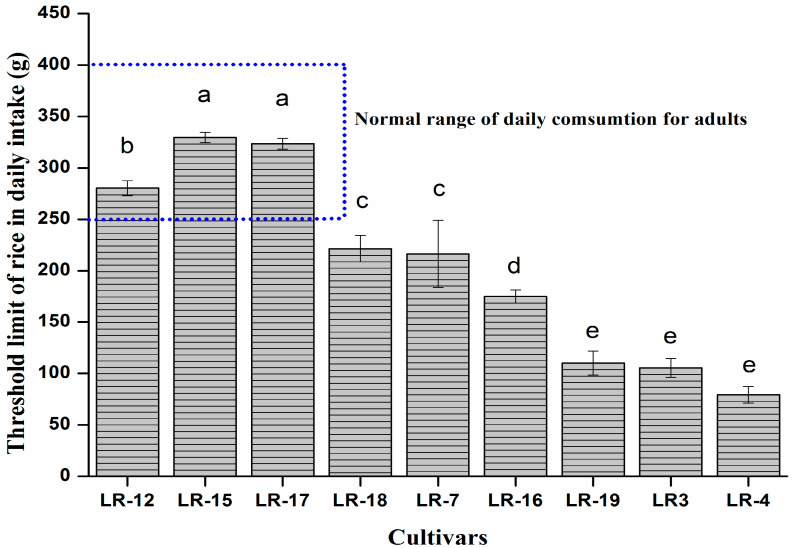
Daily dietary threshold limit of late rice grain on the basis of Cd concentration in polished rice.

Cadmium is a hazardous metal for human health as its toxicity can impair susceptible organs (kidneys). Therefore, it is necessary to minimize human exposure and decrease potential health risks associated with Cd ingestion through food. Estimated daily intake (EDI) of Cd ingestion was calculated on the basis of daily consumption 0.25–0.4 kg (Figure 8) and then divided by 70 kg (an average human body weight) [50,51]. The maximum permissible limit of Cd intake set by Joint FAO/WHO Expert Committee on Food Additives (JECFA) [52] is 0.25 μg kg^−1^, so we calculated the daily dietary intake of polished rice on the basis of the maximum threshold limit (Figure 8).

We selected nine cultivars for the 2nd year experiment to verify the 1st year’s results. On the basis of Cd accumulation in polished rice only three cultivars (LR-12, LR-15 and LR-17) were in the normal range of daily intake. While LR-15 and LR-17 polished rice consumption on behalf of Cd concentration were less than the provisional tolerable daily intake (PTDI) recommended by the JECFA [52], which are found safe for daily dietary consumption of polished rice up to 330 and 325 g, respectively. Meanwhile, the remaining cultivars were below the normal range of daily intake by the Chinese people. Among all the cultivars, the LR-4 showed the lowest quantity (75 g) for Cd safe daily dietary intake.

### 3.8. Phytic Acid, Protein Content and Mineral Nutrient Concentration in Polished Rice

There was significant differences in nutrient concentration among the prescreened cultivars and selected for the second year experiment (Table 2). Iron (Fe) concentration in polished rice grains varied from 22.65 (LR-4) to 12.39 mg kg^−1^ (LR-18), while LR-17 had the highest concentration of manganese (Mn) and zinc (Zn; 34.18 and 26.86 mg kg^−1^, respectively). It was noteworthy that LR-16 and LR-7 showed the lowest Mn and Zn concentration in grains (13.33 and 16.34 mg kg^−1^, respectively). Cu concentration in polished rice ranged from 3.21 (LR-17) to 2.27 mg kg^−1^ (LR-7) among the cultivars. Growth and development of plants depended upon the availability of macro as well as micronutrients [53,54]. So, various interactions occurred to nutrients during the uptake and accumulation processes [55]. Phloem sap is considered the nearest source of mineral elements for the developing grains. The variations between trace metals and mineral elements demonstrate the selectivity of grains for mineral elements, and thus, there is a significant difference in grain mineral nutrient concentrations among the rice cultivars.

Phytic acid and protein contents varied in the high, medium, and low accumulator cultivar (Table 2). Phytic acid in polished rice grains ranged from 3.62 in LR-3 to 1.26 mg g^−1^ in LR-7. The results from the present study showed that cultivars differed in phytic acid contents, which may affect Cd concentration in polished rice. Low phytic acid contents in cereals are considered important for the bioaccessibility of mineral nutrients. Egli [56] stated that the lesser phytate concentration in grains might lead to the elevated mineral contents (Zn and Fe). Some other studies also revealed that low concentration of phytate might increase the assimilation of Zn and Fe [57,58].

## 4. Conclusion

In this study, we conducted two-year experiments to screen low Cd accumulator late rice, by evaluating 27 cultivars in the first year proceeded by 9 cultivars as low, medium and high accumulators to verify in the 2nd year experiment. Five genotypes (LR-12, LR-17, LR-15, LR-18, LR-25 and LR-26) were identified as a low Cd accumulator with high mineral nutrient concentration and among them LR-12, LR-15 and LR-17 were evaluated as safe cultivars and cultivated again in the 2nd year for verification. Based on the verification results the two cultivars, i.e., LR-15 and LR-17, were considered suitable for cultivation on slightly or moderately Cd contaminated soil owing to their low Cd accumulation in grain, minimal health risk and relatively high nutrient concentrations in polished rice. These two cultivars also fulfilled the threshold limit for human consumption based on Cd dietary intake in the respective area. Screening of low Cd accumulator cultivars is a feasible and appropriate approach for safe production of rice as conventional breeding methods for Cd tolerance cultivars is often time consuming and costly. However, further research is needed to understand the metal uptake and accumulation mechanisms, with different soil types, interactions between Cd and mineral nutrients for the safe production of nutritive crops.

## Figures and Tables

**Table 1:** Basic physio-chemical properties of the soil used for the experiment.

Properties	Values
Soil type	Red earth
pH	5.83 ± 0.04
Organic matter (%)	7.25 ± 0.03
Total N (g kg^−1^)	2.10 ± 0.03
Available P (mg kg^−1^)	53.9 ± 4.69
Available K (mg kg^−1^)	120 ± 9.8
Total Cd (mg kg^−1^)	0.85 ± 0.06
Available Cd (mg kg^−1^)	0.34 ± 0.02

**Table 2:** Grain Cd concentration, grain yield and mineral nutrient concentration in polished rice of cultivars in the 2nd year experiment. Data are means of three replicates; followed by different letters indicate significant difference *p* < 0.05. MID and HIGH represent medium and high Cd accumulation cultivars in the first-year experiment.

Type	Cultivars	Cd Conc. (mg kg^−1^)	Grains Yield (kg Mu^−1^)	Cu (mg kg^−1^)	Fe (mg kg^−1^)	Mn (mg kg^−1^)	Zn (mg kg^−1^)	Protein %	Phytic Acid
LOW	LR-12	0.20 ± 0.00 d	486.36 ± 0.94 b	2.32 ± 0.08 e	12.5 ± 1.71 d	16.03 ± 0.22 ef	18.73 ± 0.23 cd	9.42 ± 0.07 c	2.89 ± 0.06 b
	LR-15	0.17 ± 0.00 d	558.67 ± 1.11 a	2.93 ± 0.06 bc	14.29 ± 0.24 d	26.24 ± 1.52 bc	23.67 ± 0.42 b	10.17 ± 0.08 b	1.50 ± 0.33 cd
	LR-17	0.18 ± 0.00 d	395.02 ± 1.04 g	3.21 ± 0.11 a	22.65 ± 0.99 a	34.18 ± 1.71 a	26.86 ± 1.03 a	8.42 ± 0.25 d	1.64 ± 0.17 cd
MID	LR-18	0.26 ± 0.01 cd	449.63 ± 6.29 c	2.63 ± 0.04 d	12.39 ± 0.35 d	15.24 ± 0.08 ef	16.76 ± 0.02 de	8.70 ± 0.03 d	1.31 ± 0.11 d
	LR-7	0.28 ± 0.03 cd	436.14 ± 0.86 d	2.27 ± 0.03 e	17.60 ± 0.42 c	18.61 ± 0.21 def	16.34 ± 0.15 e	8.79 ± 0.08 d	1.26 ± 0.25 d
	LR-16	0.33 ± 0.01 c	413.69 ± 1.79 f	2.28 ± 0.04 e	12.63 ± 1.72 d	13.33 ± 0.18 f	17.41 ± 0.41 cde	8.02 ± 0.14 e	1.44 ± 0.06 cd
HIGH	LR-19	0.54 ± 0.05 b	409.92 ± 0.09 f	2.73 ± 0.06 cd	18.58 ± 0.94 bc	29.80 ± 5.15 ab	19.14 ± 1.4 c	10.01 ± 0.07 b	3.53 ± 0.13 a
	LR3	0.56 ± 0.05 b	426.84 ± 0.11 e	3.06 ± 0.11 ab	20.98 ± 1.29 ab	24.44 ± 0.03 bcd	17.91 ± 0.08 cde	10.66 ± 0.03 a	3.62 ± 0.11 a
	LR-4	0.74 ± 0.06 a	492.56 ± 0.85 b	2.82 ± 0.05 cd	22.70 ± 0.87 a	21.26 ± 1.43 cde	17.39 ± 0.7 cde	7.75 ± 0.15 e	1.94 ± 0.06 c

## Supplementary Materials

The following are available online at https://www.mdpi.com/1660-4601/17/10/3632/s1, Table S1. Cultivars information tested for the Cd uptake, accumulation and health risk assessment.
