# Supplementary material for: Cataloging of Cd Allocation in Late Rice Cultivars Grown in Polluted Gleysol: Implications for Selection of Cultivars with Minimal Risk to Human Health
Source: Int J Environ Res Public Health. 2020 May 21;17(10):3632. doi: 10.3390/ijerph17103632 (PMC7277880; doi:10.3390/ijerph17103632)
Supplement: Supplementary file 1 [file ijerph-17-03632-s001.pdf]

Table S1. Cultivars information tested for the Cd uptake, accumulation and health risk assessment

Cultivar no	Cultivar name	Cultivar Type	Cultivar no	Cultivar name	Cultivar Type
LR-1	shenliangyou5814	Indica Two-line Hybrid Rice	LR-15	wuyou103	Indica Three-line Hybrid Rice
LR-2	liangyou336	Indica Two-line Hybrid Rice	LR-16	yueyou9133	Indica Three-line Hybrid Rice
LR-3	taiyou398	Indica Three-line Hybrid Rice	LR-17	wushanshimiao	Conventional Rice
LR-4	wuyouhuazhan	Indica Three-line Hybrid Rice	LR-18	Hyou158	Indica Two-line Hybrid Rice
LR-5	taiyou98	Indica Three-line Hybrid Rice	LR-19	shenyou9566	Indica Two-line Hybrid Rice
LR-6	zaofengyouhuazhan	Indica Three-line Hybrid Rice	LR-20	xinliangyou611	Indica Two-line Hybrid Rice
LR-7	wuyou61	Indica Three-line Hybrid Rice	LR-21	liangyou6026	Indica Two-line Hybrid Rice
LR-8	jiyouyazhan	Indica Three-line Hybrid Rice	LR-22	fengliangyouwansan	Indica Two-line Hybrid Rice
LR-9	rongyouhuazhan	Indica Three-line Hybrid Rice	LR-23	zhunliangyou893	Indica Two-line Hybrid Rice
LR-10	tianyouyazhan	Indica Three-line Hybrid Rice	LR-24	SM727	Japonica Conventional Rice
LR-11	meixiangxinzhan	Indica Conventional Rice	LR-25	xidaosanhao	Japonica Conventional Rice
LR-12	Hyou518	Indica Three-line Hybrid Rice	LR-26	SM728	Japonica Conventional Rice
LR-13	wuyou308	Indica Three-line Hybrid Rice	LR-27	yonyoujiuhao	Japonica Three-line Hybrid Rice
LR-14	rongxiangyouhuazhan	Indica Three-line Hybrid Rice			
